# The value-based price of transformative gene therapy for sickle cell disease: a modeling analysis

**DOI:** 10.1038/s41598-024-53121-0

**Published:** 2024-02-01

**Authors:** George Morgan, Emily Back, Martin Besser, Timothy B. Hallett, Gregory F. Guzauskas

**Affiliations:** 1 Prime HCD, Mere House, Brook St, Knutsford, WA16 8GP UK; 2 Departments of Haematology, Addenbrooke’s Hospital, Cambridge, UK; 3 MRC Centre for Global Infectious Disease Analysis, Imperial College London, London, UK; 4 The Comparative Health Outcomes, Policy, and Economics Institute, University of Washington, Seattle, WA USA

**Keywords:** Health care economics, Sickle cell disease

## Abstract

Sickle cell disease (SCD) is an inherited, progressively debilitating blood disorder. Emerging gene therapies (GTx) may lead to a complete remission, the benefits of such can only be realized if GTx is affordable and accessible in the low-and middle-income countries (LMIC) with the greatest SCD burden. To estimate the health impacts and country-specific value-based prices (VBP) of a future gene therapy for SCD using a cost-utility model framework. We developed a lifetime Markov model to compare the costs and health outcomes of GTx versus standard of care for SCD. We modeled populations in seven LMICs and six high-income countries (HICs) estimating lifetime costs and disability-adjusted life-years (DALYs) in comparison to estimates of a country’s cost-effectiveness threshold. Each country’s unique VBP for GTx was calculated via threshold analysis. Relative to SOC treatment alone, we found that hypothetical GTx reduced the number of people symptomatic with SCD over time leading to fewer DALYs. Across countries, VBPs ranged from $3.6 million (US) to $700 (Uganda). Our results indicate a wide range of GTx prices are required if it is to be made widely available and may inform burden and affordability for ‘target product profiles’ of GTx in SCD.

## Introduction

Sickle cell disease (SCD) is an inherited disorder resulting from an autosomal recessive mutation of the gene that encodes the beta-globin protein and is characterized by a structural abnormality in red blood cells^1^. SCD can lead to clinical manifestations such as haemolytic anaemia, recurrent episodes of vaso-occlusive crisis (VOC), inflammation, and severe pain, which manifest with increasing age. The disease is progressively debilitating, resulting in increased healthcare resource utilization and reduced life expectancy^2,3^.

Over 500,000 infants are born with SCD every year^4^, predominantly in low- and middle-income countries (LMIC). Severe disease and higher prevalence tend to be predominant in sub-Saharan and northeast Africa, India, and the Middle East^4–6^. Furthermore, LMICs tend to lack national newborn screening and modern standard of care (SOC) programs that would minimize SCD morbidity and mortality via early diagnosis, targeted vaccinations, regular check-ups, treatment management, and parental education^7^. In high-income countries (HIC), strong evidence supports the impact of early diagnosis and associated care in countering the increased risk of morbidity and mortality from acute VOC events^8,9^.

The current treatment landscape for SCD is varied, with hydroxyurea commonly used in HICs to reduce the frequency of acute VOC and acute chest syndrome^10,11^ but with limited use in LMICs due to financial inaccessibility and scarce availability^12–14^. Other regimens recently evaluated by the US Food and Drug Administration (including crizanlizumab, voxelotor, and l-glutamine) have shown additional benefit in the reduction of acute pain episodes and hospitalisations compared to current SOC, however, real-world data and LMIC access at current prices are lacking^11,15^. Lastly, haematopoietic stem cell transplantation is a potentially transformative procedure^12,13^ and in the UK (subject to approval by a national panel), the National Health Service has decided to fund sibling allografts for severe SCD. However, obstacles to this procedure’s widespread uptake include a scarcity of suitable donors, serious adverse events, and high cost.

The FDA recently approved^16^ the gene therapies (GTx) Lyfgenia, which produces an anti-sickling hemoglobin (HbA^T87Q^) using a one-time treatment^17^, and Casgevy, a cell-based gene therapy that utilizes CRISPR-Cas9 and is designed to compensate for the loss of hemoglobin by inducing fetal hemoglobin^18^. Initial clinical trial results have indicated a sustained production of HbA^T87^^Q^ and fetal hemoglobin expression surges for Lyfgenia and Casgevy, respectively, leading to complete resolution of severe VOC episodes, though their long-term efficacy and safety remain uncertain^18,19^.

The potential for complete remission of SCD via GTx raises complex questions around the pricing mechanism of a one-time administration treatment intervention. Solutions to these complex questions require evidence to outline the economic value of a GTx in SCD in different markets. An optimal pricing mechanism for such therapies could be based on the ability and willingness of a given country to pay for improved population health; using this approach, each country would set its cost-effectiveness thresholds based on its marginal opportunity costs^20^. If the price of an intervention is up to or below these thresholds, the resulting price and utilization would be “value-based”^21,22^.

Given the disproportionate SCD burden and inadequate access to available interventions in LMICs, further understanding of the pricing mechanism for transformative GTx is required^23,24^. Our first objective was to develop a decision-analytic model that reflects the current ability of selected countries to provide newborn screening to forecast long-term SCD-related costs and health outcomes. Our second objective was to estimate the value-based price (VBP) appropriate for each country to pay per GTx administration based on their unique cost-effectiveness thresholds^12^.

## Methods

### Modeling overview

Decision-analytic models are widely employed to estimate the costs, outcomes, and cost-effectiveness of treatments, and state-transition (or Markov) modeling specifically is one of the most widely used computational modeling approaches. Markov models utilize health states and time-dependent transitions among these states to represent the natural history of a disease and the impacts of treatment over a period of time^25,26^. Specifically, proportions of a hypothetical cohort reside in each health state per model cycle of a specified duration and transition among health states in subsequent cycles.

The proportion of individuals in each health state per cycle can be used with state values (e.g., life-years, health state-specific disability weights, and costs) to estimate life expectancy, disability-adjusted life years (DALYs), and expected costs, respectively^25,26^. DALYs are the measure of population health estimated over the modeled time horizon, calculated as the sum of the years of life lost due to premature mortality (YLLs) from SCD and the years of healthy life lost due to SCD-associated disability (YLDs)^27^. To calculate YLD, the number of people in each health state per model cycle is multiplied by a disability weight, with perfect health having a weight of 0, 1 equates to death, and values in between represent varying degrees of morbidity.

The incremental cost-effectiveness ratio (ICER) is calculated as the difference in the cost between modeled comparators divided by the difference in their effectiveness (as measured in DALYs) to give the cost per DALY averted. The incremental cost-effectiveness ratio threshold for each country is the benchmark DALYs averted per unit cost that a new intervention must exceed to be “cost-effective”. Knowing these thresholds allows for the calculation of the VBP, which is the maximum each country’s health system should pay for an intervention with a given health benefit. This model uses the accepted incremental cost-effectiveness ratio thresholds for HICs and a framework from a study by Ochalek et al.^28^ for generating LMIC-specific threshold estimates.

### SCD state transition model

Our Markov model was designed to assess the cost-effectiveness of a hypothetical GTx for SCD from a multi-country perspective. We modeled seven LMICs (Ghana, India, Kenya, Nigeria, South Africa, Uganda, and Zambia) and six HICs (France, Germany, Italy, Spain, the United Kingdom, and the United States).The model was developed in tandem with a related exploratory study on the value of future GTx for HIV^29^; as such, the included LMICs were deliberately selected for their high disease burdens of one or both diseases, with plans to add additional countries in future model adaptations.

Where possible, we used available data on each country’s newborn screening rates and economic factors from global databases and published literature (Table 1). We estimated the incremental cost, life years, and DALYs resulting from a novel GTx alongside SOC versus the use of SOC as the stand-alone treatment option. We utilised annual model cycles and a lifetime time horizon to account for differences in cost and health outcomes that persist for the remainder of modeled individuals’ lives. Furthermore, we assumed a societal perspective and discounted all future cost and health outcomes by 3% per year to reflect their present value^30^. The model was developed in Microsoft^®^ Excel^®^.

**Table 1:** Gene Therapy and SOC Scenarios Parameters.

Gene therapy assumptions (all countries)	Pessimistic GTx complete remission scenario		Base case GTx complete remission scenario		Optimistic GTx complete remission scenario		Newborn screening scenario (LMIC)		Quality of SOC scenario (LMIC)	
	LMIC	HIC	LMIC	HIC	LMIC	HIC	LMIC 1	LMIC 2	LMIC 1	LMIC 2
Remission probability	35%	35%	65%	65%	95%	95%	65%	65%	65%	65%
Remission durability (years)	5	5	10	10	Lifetime	Lifetime	10	10	10	10
Eligibility: latest disease stage	Mild	Mild	Severe	Severe	Severe	Severe	Severe	Severe	Severe	Severe
Eligibility: age	18	18	12	12	0	0	12	12	12	12
Eligibility: repeat GTx administration after relapse	No	No	No	No	Yes	Yes	No	No	No	No
Maximum uptake proportion/year	25%	25%	50%	50%	75%	75%	50%	50%	50%	50%
Years until maximum uptake achieved	15	15	10	10	5	5	10	10	10	10
Standard of care assumptions										
Newborn screening rate	5%	97%	5%	97%	5%	97%	25%	50%	5%	5%
Standard of care multiplier	0.05	NA	0.05	NA	0.05	NA	0.05	0.05	NA	0.10

*GTx* gene therapy, *LMIC* low-middle income country, *HIC* high income country, *SOC* standard of care.

Newborn screening (the percentage of the population screened at birth), SOC multiplier (the quality of standard of care effect on transition probabilities), effectiveness (the per individual probability that gene therapy successfully treats SCD), eligibility (latest disease stage in which affected individuals remain eligible for GTx), uptake (the maximum expected annual percentage of those with SCD who receive GTx).

**Figure 1:**
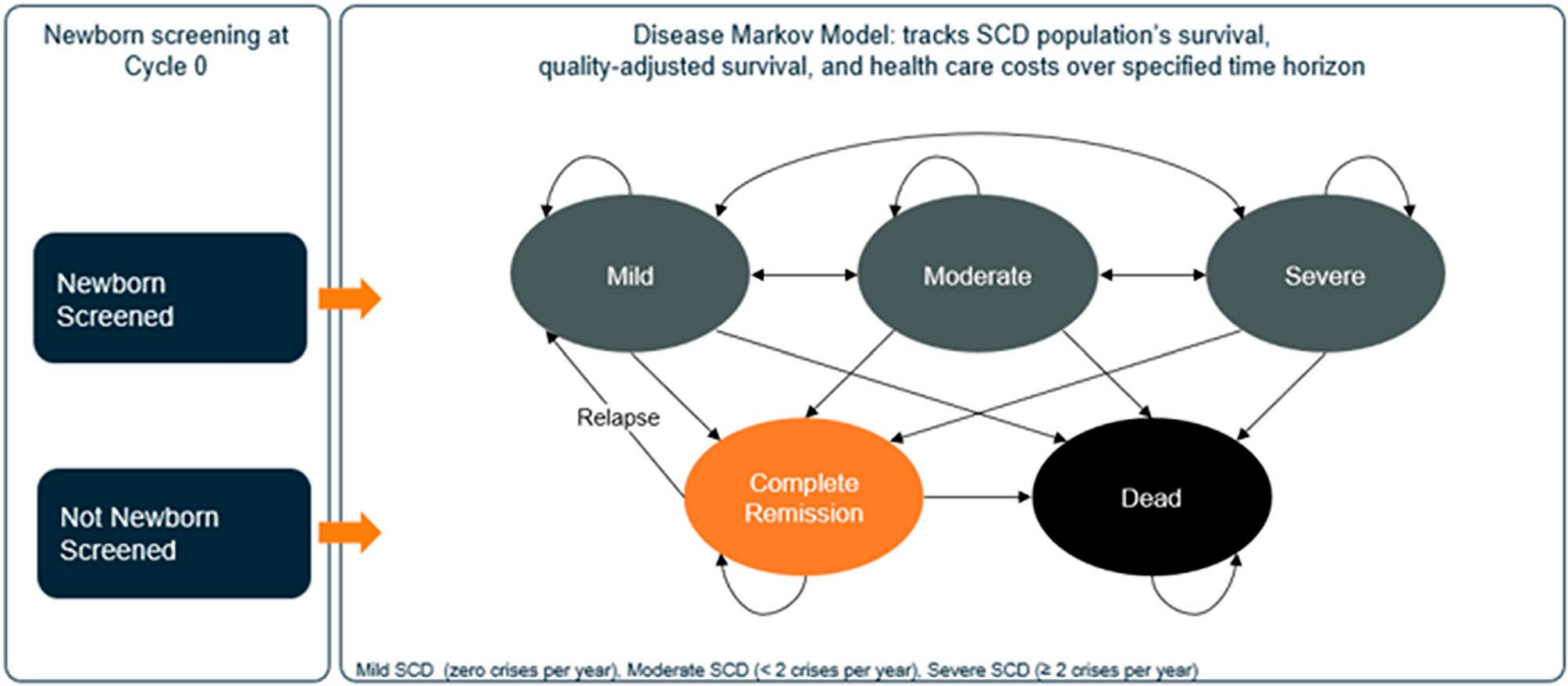
SCD model schematic. *SCD* sickle cell disease, ‘newborn screened’ enter the model with a lower mortality rate compared to ‘not newborn screened’.

Hypothetical individuals with SCD entered the model in the year 2030, which we assume as the year for a global rollout of GTx. A country-specific proportion of these individuals received early SCD diagnosis via newborn screening. Those diagnosed early with SCD had a decreased risk of death from SCD complications due to access to preventive measures such as pneumococcal vaccination and prophylactic penicillin. Affected individuals were distributed among the following health states: mild SCD (asymptomatic, newborn screened or not), moderate SCD (1–2 VOC/year, screened or not), and severe SCD (≥ 3 VOC/year, screened or not) in the first model cycle (Fig. 1)^11^. In subsequent cycles, modeled individuals could transition with age-based, mortality-conditional transition probabilities (Appendix Figure A1–A5) to better or worse SCD health states, remain in the previous cycle health state, or die; death was an absorbing state, i.e., it is impossible to transition from that state, and could occur via SCD-associated mortality or background mortality^31^. In the GTx comparator scenario, individuals receive SOC until they reach eligibility age, at which point they could receive transformative GTx, after which they were assumed to reside in the complete remission (asymptomatic and cost-free) health state until they either relapsed or died. Those who relapsed and**/**or whose treatment failed moved back into the mild health state where they could then transition to a moderate or severe health state unless they received repeated GTx (in a scenario analysis) and moved back to the complete remission state.

Of note, management and treatment strategies vary greatly by clinic location, age, and severity, as well as across and within countries. To account for the variability of SOC in SCD, a combination of treatment options have to be defined. Our model’s transition probabilities were derived from Salcedo et al. (2021), hence our SOC is defined as antibiotics, vaccinations, pain-relief medications, hydroxyurea, blood transfusions, and/or stem cell transplants^11^. We did not differentiate individuals within the SOC group based on specific treatments received. No individuals with SCD were involved in the process of this research.

### Model parameters

The underlying epidemiological and cost parameters are provided in the Appendix (Table A5). We modelled a newborn screening proportion of 5% for LMICs based on the available literature including the CONSA screening programme, which aims to screen 10,000–16,000 newborns per year^32,33^. For HICs, a 97% newborn screening proportion was used^8^. Those not diagnosed via newborn screening had a higher mortality but still received SOC. The initial distribution among health states and subsequent transition probabilities was obtained from a previous US-based cost-effectiveness analysis^11^. Mortality was estimated using annualised estimates obtained from Runkel et al., who extracted the mortality benefits of newborn screening for SCD followed by an earlier ameliorative treatment start, showing the effectiveness of SOC in reducing the case fatality rate for SCD^31^. We utilized health state-specific disability weights from the Global Burden of Disease study, which surveyed > 60,000 respondents aged 18–65 to provide quantitative estimates of health losses associated with non-fatal outcomes in 183 health states including anaemia^34^.

In the calculation of SOC, USA-based treatment costs were obtained from Gallagher et al.^35^ who published SCD related costs across a 5-year period for a Medicare population. These 5 year average costs were separated into five categories; outpatient pharmacy, other outpatient services, outpatient visits, emergency room, and inpatient. We assumed the mild health state SOC cost consisted of outpatient pharmacy, other outpatient services, and outpatient visit, which was equivalent to the standard of care cost in the ICER 2023 review^36^. The moderate health state SOC cost was assumed to consist of outpatient pharmacy, other outpatient services, outpatient visit, plus a 16.5% proportion of the emergency room and inpatient costs; this proportion was derived via the Medicare cohort, which had an average of 45.5 VOC’s over 5 years versus the moderate SCD estimate of 1–2 VOC’s per year (16.5% × 45.5 = 1.5). We assumed severe health state costs consisted of all five categories.

Non-US-based SOC costs for each health state were largely unavailable, necessitating assumed values. We used the US-based health state costs described above to derive estimates for the other countries by weighting them by their relative health expenditure compared to the US (Table A1–A3 of the Appendix)^34^. We validated our approach for assuming non-US SOC cost values by comparing similarly derived estimates for Congo (LIC) to the average SCD costs reported in a recent publication (Table A4 of the Appendix)^37^, and found that our derived estimates were similar (mild ± 37%, moderate ± 29%, severe ± 16%).

The cost of GTx was not explicitly modelled as an input parameter but, rather, as an output dependent on each country’s willingness-to-pay per DALY averted threshold. Societal costs were calculated using the productivity percentage lost (51.7% for mild and moderate SCD states and 62.1% for the severe state) in terms of average annual salary for each country, based on statistics reported by Rizio et al.^38^; these authors explored the impact of VOC on HRQoL and work productivity to find more significant deficits in HRQoL and work productivity when individuals with SCD were stratified by VOC severity and frequency. The model parameters can be found in Table A5 in the Appendix.

### Structural assumptions

In the GTx comparator, we assumed that eligible individuals age ≥ 12 with SCD could achieve complete remission with a single dose of a hypothetical GTx regardless of SCD severity. We considered lifetime durability of the complete remission being dependent on the likelihood of whether SCD relapse occurs or not. Post-relapse individuals were ineligible for a repeated GTx administration in the base case analysis. We assumed a maximum GTx uptake by eligible individuals per year of 50% and a linear scale-up to the maximum uptake over time.

We explored multiple inherent uncertainties regarding the efficacy, durability, and eligibility of a hypothetical GTx compared to SOC via three scenario analysis combinations of parameter assumptions: base case (neutral), pessimistic, and optimistic (Table 1). These scenarios were applied to all thirteen countries. We also assumed that LMICs provided a lower quality of SOC, in terms of the difference in the accessibility and standards of treatment options on offer, when compared to HICs and this was reflected in the model via a quality of SOC multiplier that increased the annual transition probabilities for moderate and severe SCD in LMICs by up to 10%.

### Model analysis

Our health economic decision-analytic model allows for the calibration of variables such as the remission probability, durability of the GTx treatment effect, and uptake over time. This model can provide estimates for life years gained, SCD deaths prevented, DALYs averted, and the incremental costs. These estimates are calculated for each of the thirteen countries using modular, variable assumptions for GTx impact alongside potential SOC scenarios. We then computed the VBP for all thirteen countries and how these VBP’s were affected by the properties of the hypothetical GTx and the base case, pessimistic, and optimistic scenarios.

**Figure 2:**
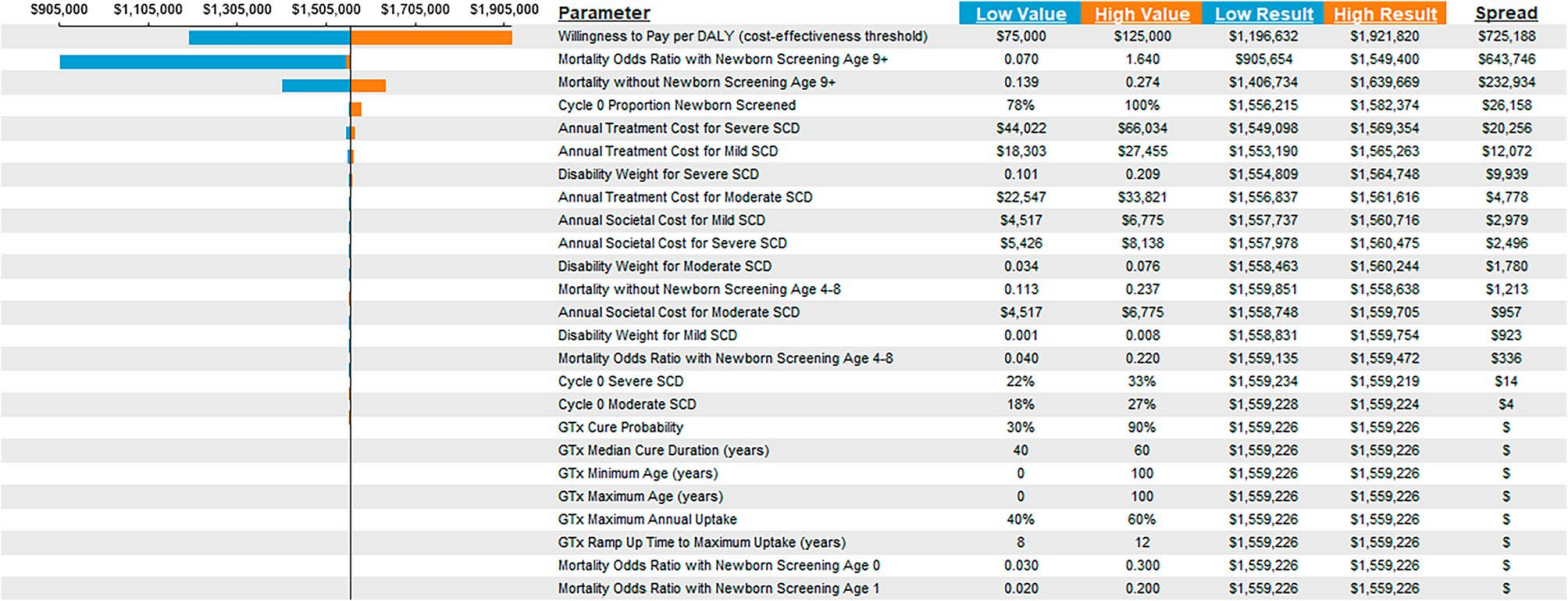
Tornado diagram ranking the parameters that estimate uncertainty in the VBP outcome for US. *SCD* sickle cell disease*.*

We performed a scenario analysis by varying the newborn screening rate for LMICs from 5 to 25% and 50%, thereby capturing the impact that an increased screening rate has on survival and other outcomes of the model^11^. In another scenario analysis to test the impact of changes in the provision of SOC in LMICs, we set the SOC multiplier (base case 5% in LMICs) to 0%, and 10% relative increases in the probability of disease progression. We also performed one-way sensitivity analyses to assess the impacts of parameter uncertainty on the model’s VBP results; tornado diagrams, which visually rank the parameters whose estimate uncertainty has the greatest impacts on the VBP, were produced for each country case study (Fig. 2 and Figs. A6–A17).

## Results

In the base case scenario we estimated that the introduction of GTx starting in the year 2030 reduced (a) the number of individuals symptomatic with SCD, therefore increasing life years (from 0.86 years in South Africa to 1.36 in Italy) and (b) the number of individuals dying from SCD, leading to fewer DALYs than SOC treatment alone. Incremental DALYs averted ranged from 1.7 in Nigeria to 5.76 in Italy. Over the time horizon, non-GTx costs ranged from $230 (rounded) in Uganda to $550,700 (rounded) in the USA. In the pessimistic scenario, total life years ranged from 0.01 years in South Africa to 0.43 years in France, Germany, UK and USA, with DALYs averted ranging from 0.09 in Nigeria to 0.71 in Italy. Incremental total cost ranged from $12 in Uganda to $67,300 (rounded) in the USA. In the optimistic scenario, life years ranged from 10.40 years in South Africa to 13.39 years in Italy, with DALYS averted ranging from 19.06 in Nigeria to 30.53 in Italy. Incremental total cost ranged from $2,600 (rounded) in Uganda to $3 million (rounded) in the USA. The outcomes by country and scenario are presented in Table 2.

**Table 2:** Outcomes by country and scenario.

Countries by scenario		Total life years			Total DALYs			Total cost		
		GTx	SOC	Life years gained	GTx	SOC	DALYs averted	GTx	SOC	Incremental cost
Ghana	Pessimistic	3.99	3.97	**0.02**	40.11	40.21	**0.10**	$1133	$1076	**$57**
	Base case	4.86	3.97	**0.89**	38.28	40.21	**1.93**	$2141	$1076	**$1065**
	Optimistic	14.70	3.97	**10.73**	18.32	40.21	**21.89**	$13,160	$1076	**$12,084**
	NBS 25%	5.93	4.97	**0.95**	35.28	37.78	**2.49**	$2717	$1341	**$1376**
	NBS 50%	7.26	6.23	**1.03**	31.54	34.74	**3.20**	$3437	$1673	**$1765**
	SOCM 0%	4.86	3.97	**0.89**	38.27	40.20	**1.93**	$2126	$1062	**$1064**
	SOCM 10%	4.86	3.97	**0.89**	38.29	40.22	**1.93**	$2156	$1091	**$1065**
India	Pessimistic	3.99	3.97	**0.02**	38.66	38.76	**0.10**	$958	$918	**$40**
	Base case	4.88	3.97	**0.91**	36.87	38.76	**1.89**	$1669	$918	**$750**
	Optimistic	15.04	3.97	**11.07**	17.24	38.76	**21.52**	$9464	$918	**$8545**
	NBS 25%	5.96	4.97	**0.98**	33.95	36.39	**2.45**	$2116	$1145	**$971**
	NBS 50%	7.30	6.23	**1.07**	30.29	33.43	**3.14**	$2675	$1427	**$1247**
	SOCM 0%	4.88	3.97	**0.91**	36.86	38.75	**1.89**	$1656	$906	**$750**
	SOCM 10%	4.88	3.97	**0.91**	36.88	38.77	**1.89**	$1682	$931	**$751**
Kenya	Pessimistic	3.99	3.97	**0.02**	40.32	40.43	**0.10**	$1241	$1191	**$50**
	Base case	4.86	3.97	**0.89**	38.49	40.43	**1.93**	$2125	$1191	**$933**
	Optimistic	14.65	3.97	**10.68**	18.50	40.43	**21.92**	$11,779	$1191	**$10,588**
	NBS 25%	5.92	4.97	**0.95**	35.49	37.98	**2.50**	$2690	$1484	**$1206**
	NBS 50%	7.25	6.23	**1.02**	31.73	34.93	**3.20**	$3397	$1851	**$1546**
	SOCM 0%	4.86	3.97	**0.89**	38.48	40.42	**1.93**	$2108	$1175	**$933**
	SOCM 10%	4.86	3.97	**0.89**	38.50	40.44	**1.93**	$2141	£1208	**$934**
Nigeria	Pessimistic	3.99	3.97	**0.02**	34.70	34.79	**0.09**	$1039	$1019	**$20**
	Base	4.87	3.97	**0.90**	33.10	34.79	**1.70**	$1398	$1019	**$379**
	Optimistic	14.79	3.97	**10.82**	15.73	34.79	**19.06**	$5269	$1019	**$4251**
	NBS 25%	5.94	4.97	**0.97**	30.41	32.60	**2.19**	$1758	$1270	**$488**
	NBS 50%	7.28	6.23	**1.05**	27.05	29.85	**2.80**	$2209	$1583	**$625**
	SOCM 0%	4.87	3.97	**0.90**	33.09	34.78	**1.70**	$1384	$1005	**$379**
	SOCM 10%	4.87	3.97	**0.90**	33.10	34.80	**1.70**	$1412	$1033	**$379**
South Africa	Pessimistic	3.98	3.97	**0.01**	40.08	40.18	**0.10**	$8169	$7850	**$319**
	Base case	4.83	3.97	**0.86**	38.30	40.18	**1.88**	$13,906	$7850	**$6056**
	Optimistic	14.37	3.97	**10.40**	18.85	40.18	**21.32**	$76,687	$7850	**$68,837**
	NBS 25%	5.88	4.97	**0.91**	35.33	37.74	**2.41**	$17,570	$9783	**$7787**
	NBS 50%	7.19	6.23	**0.97**	31.62	34.70	**3.08**	$22,150	$12,199	**$9951**
	SOCM 0%	4.83	3.97	**0.86**	38.29	40.17	**1.88**	$13,799	$7745	**$6054**
	SOCM 10%	4.83	3.97	**0.86**	38.31	40.19	**1.88**	$14,017	$7959	**$6058**
Uganda	Pessimistic	3.99	3.97	**0.02**	39.83	39.93	**0.10**	$471	$459	**$12**
	Base case	4.86	3.97	**0.89**	38.02	39.93	**1.92**	$689	$459	**$230**
	Optimistic	14.71	3.97	**10.74**	18.19	39.93	**21.74**	$3068	$459	**$2609**
	NBS 25%	5.93	4.97	**0.96**	35.03	37.51	**2.48**	$870	$572	**$297**
	NBS 50%	7.26	6.23	**1.03**	31.31	34.49	**3.18**	$1095	$714	**$381**
	SOCM 0%	4.86	3.97	**0.89**	38.01	39.92	**1.92**	$683	$453	**$230**
	SOCM 10%	4.86	3.97	**0.89**	38.03	39.94	**1.92**	$696	$466	**$230**
Zambia	Pessimistic	3.99	3.97	**0.02**	38.02	38.12	**0.10**	$1047	$990	**$57**
	Base case	4.84	3.97	**0.87**	36.30	38.12	**1.81**	$2061	$990	**$1071**
	Optimistic	14.41	3.97	**10.44**	17.62	38.12	**20.50**	$13,104	$990	**$12,114**
	NBS 25%	5.90	4.97	**0.93**	33.44	35.77	**2.33**	$2613	$1234	**$1379**
	NBS 50%	7.22	6.23	**0.99**	29.86	32.85	**2.99**	$3303	$1539	**$1764**
	SOCM 0%	4.84	3.97	**0.87**	36.29	38.11	**1.81**	$2048	$977	**$1071**
	SOCM 10%	4.84	3.97	**0.87**	36.31	38.12	**1.81**	$2075	$1004	**$1071**
France	Pessimistic	8.16	8.58	**0.43**	35.42	36.12	**0.70**	$173,884	$135,238	**$38,646**
	Base case	9.93	8.58	**1.34**	30.38	36.12	**5.74**	$450,928	$135,238	**$315,691**
	Optimistic	21.89	8.58	**13.30**	5.74	36.12	**30.38**	$1,807,035	$135,238	**$1,671,797**
Germany	Pessimistic	8.16	8.58	**0.43**	34.84	35.54	**0.69**	$224,742	$163,778	**$60,964**
	Base case	9.93	8.58	**1.34**	29.88	35.54	**5.66**	$661,733	$163,778	**$497,955**
	Optimistic	21.81	8.58	**13.22**	5.65	35.54	**29.89**	$2,794,945	$163,778	**$2,631,167**
Italy	Pessimistic	8.16	8.58	**0.42**	35.41	36.12	**0.71**	$122,761	$87,489	**$35,272**
	Base case	9.94	8.58	**1.36**	30.36	36.12	**5.76**	$375,376	$87,489	**$287,887**
	Optimistic	21.97	8.58	**13.39**	5.59	36.12	**30.53**	$1,614,089	$87,489	**$1,526,601**
Spain	Pessimistic	8.16	8.58	**0.42**	35.39	36.10	**0.70**	$104,888	$81,618	**$23,270**
	Base case	9.94	8.58	**1.35**	30.34	36.10	**5.75**	$271,553	$81,618	**$189,934**
	Optimistic	21.96	8.58	**13.38**	5.60	36.10	**30.50**	$1,088,512	$81,618	$1,006,893
UK	Pessimistic	8.16	8.58	**0.43**	35.07	35.77	**0.70**	$157,245	$129,848	**$27,397**
	Base case	9.93	8.58	**1.34**	30.08	35.77	**5.69**	$353,749	$129,848	**$223,900**
	Optimistic	21.84	8.58	**13.26**	5.68	35.77	**30.08**	$1,314,467	$129,848	**$1,184,618**
USA	Pessimistic	8.15	8.58	**0.43**	34.37	35.04	**0.67**	$396,064	$328,799	**$67,265**
	Base case	9.89	8.58	**1.30**	29.53	35.04	**5.51**	$879,464	$328,799	**$550,666**
	Optimistic	21.59	8.58	**13.00**	5.99	35.04	**29.05**	$3,233,854	$328,799	**$2,905,056**

*GTx* gene therapy, *LMIC* low-middle income country, *HIC* high income country, *SOC* standard of care, *NBS* newborn screening, the percentage of the population screened at birth, *SOCM* standard of care multiplier, the quality of standard of care effect on transition probabilities.

Significant values are in bold.

Estimates of the VBP under the three GTx scenarios are shown in Table 3 for each of the included countries. For each individual country, the VBP was higher when the efficacy and roll out of GTx was more favorable. Among countries, those with higher willingness to pay per DALY thresholds tended to have higher VBPs; across the three scenarios Uganda ($120 threshold) had the lowest VBP while the USA ($100,000 threshold) had the highest. The base case scenario saw VBPs range from $1900 (rounded) in Uganda to $1.6 million (rounded) in the USA. In the pessimistic scenario, VBPs ranged from $700 (rounded) in Uganda to $538,700 (rounded) for the USA. In the optimistic scenario, VBPs ranged from $4100 (rounded) in Uganda to $3.6 million (rounded) for USA.

**Table 3:** Value-based price of gene therapy by modeled country.

Country		Cost-effectiveness threshold	Pessimistic VBP	Base case VBP	Optimistic VBP	NBS LMIC 1 (25%)	NBS LMIC 2 (50%)	SOC multiplier scenario 1 (0%)	SOC multiplier scenario 2 (10%)
France	HIC	$55,027	$302,658	**$875,995**	$2,028,454	–	–	–	–
Germany		$88,043	$471,324	**$1,365,472**	$3,150,761	–	–	–	–
Italy		$50,000	$271,759	**$786,992**	$1,821,407	–	–	–	–
Spain		$33,016	$182,227	**$527,076**	$1,221,625	–	–	–	–
UK		$39,377	$218,762	**$632,478**	$1,466,318	–	–	–	–
USA		$100,000	$538,724	**$1,559,226**	$3,607,440	–	–	–	–
Ghana	LMIC	$552	$3073	**$8555**	$18,226	$8011	$7667	$8545	$8566
India		$397	$2167	**$6046**	$12,995	$5714	$5504	$6038	$6054
Kenya		$483	$2706	**$7537**	$16,170	$7124	$6863	$7527	$7548
Nigeria		$223	$1112	**$3128**	$6,915	$3099	$3081	$3120	$3136
South Africa		$3228	$17,432	**$48,998**	$105,402	$46,158	$44,363	$3120	$49,071
Uganda		$120	$676	**$1881**	$4,114	$1824	$1788	$48,929	$1885
Zambia		$591	$3070	**$8602**	$18,223	$8003	$7625	$8592	$8612

*GTx* gene therapy, *LMIC* low-middle income country, *HIC* high income country, *SOC* standard of care, *Newborn screening* the percentage of the population screened at birth, *SOC multiplier* the quality of standard of care effect on transition probabilities.

Significant values are in bold.

### Scenario and sensitivity analyses

The results of the scenario analysis in which newborn screening rate was varied (5% to 25% and 50%) with a base case scenario for low-middle income countries are shown in Table 3. When the rate of newborn screening was increased from 5 to 25% and 50%, the VBPs for all countries were reduced and the total life years increased. For all LMICs, increasing newborn screening from 5 to 25% and 50% resulted in increases in total incremental life years, total DALYs averted, and total incremental cost. The VBPs increased as the SOC multiplier increased from 0 to 5% and 10% due to GTx having greater value in circumstances where the SOC was worse. When we calculated the extent to which our results were uncertain due to underlying uncertainties in the parameter values, for the US we found that the model was most sensitive to the willingness to pay per DALY), the mortality odds ratio with newborn screening for ages 9+ followed by mortality odds ratio without newborn screening for ages 9+ (Fig. 2).

## Discussion

Our study investigated the potential health impacts and value of a future transformative GTx intervention for SCD in HIC and LMIC countries. Transformative GTx was found to reduce both SOC costs and SCD-associated DALYs by decreasing the risk of SCD-related complications and mortality. Across countries, VBP’s ranged from $3.6 million (USA, optimistic scenario, rounded) to $700 (Uganda, pessimistic scenario, rounded). In general, higher calculated VBPs were associated with greater GTx-conferred health benefits. Differences in the VBP among countries were primarily driven by their abilities to pay per DALY averted, and secondarily by differences in the newborn screening rates, which were explicitly linked to mortality in our model.

HIC’s and LMIC’s have notable differences in the level of SCD care they are able to provide. This dichotomy is also seen in diseases like malaria, where affected individuals in LMICs are often not appropriately diagnosed or treated in a timely manner, if at all^40^. A similar effect can be assumed for the diagnosis and treatment of SCD in these countries. Newborn screening rates for diseases like SCD also vary largely between HIC and LMICs, with more people in LMICs who are not diagnosed with SCD and therefore are not able to manage their health effectively, resulting in > 50% higher mortality rates^41^. Our newborn screening rate scenario analysis results support previous findings that serious morbidity and mortality can be prevented by early diagnosis^42^. In the context of our model for LMICs with 5% screening rate, individuals who do not receive screening have higher mortality and thus many do not live long enough to receive GTx. This limits the benefits the rollout of a future GTx could achieve if there were a higher newborn screening rate. Nevertheless, the issue of newborn screening rates must first be addressed to ensure that a majority of SCD individuals survive pre-adolescence to receive GTx treatment.

Universal access to and affordability of GTx for SCD will be challenging. Given the high manufacturing and delivery costs, there are barriers to equitable pricing in a real-world setting^43–45^. Many LMICs are unable to participate in the development and distribution of GTx due to their limited research capacity and limited advanced technology facilities^46^. Furthermore, LMIC demand for medicines appears to be more responsive to changes in the prices of medicines than is the case in HICs^47^. To assure widespread availability of life-saving GTx, this implies that pharmaceutical manufacturers need to establish their prices based on ability to pay^47^. Otherwise, LMICs will face market access restrictions, being unable to provide coverage because of the price^48^. Proposed strategies have suggested international collaborations among LMICs and HICs to identify funding solutions most appropriate for all healthcare systems^46^. Another option for pharmaceutical companies would be to explore differential pricing in each market so that access to novel therapies is maximized. Our findings include country-specific VBP for GTx from the perspective of different country-resource settings that may be helpful in the development of payment models for treatment approval in the global market, particularly in the context of LMICs. However, even at equitable prices, GTx may not be seen by decision makers as a viable option given the high burden of births and current infrastructure in LMICs. Given the current lack of availability of treatments such as hydroxyurea in these countries, these hurdles must be overcome first for transformative GTx to reach the areas of the world with the greatest need.

Previous studies have evaluated the potential benefits and financial impacts of GTx for SCD^11,24,49^. Salcedo et al. (2021)^11^ found that durable treatment would be cost-effective at a minimum willingness to pay of $150,000 per QALY at single administration costs of $713 K, $1.09 M, and $2.18 M for treatment durations of median 10 years, median 20 years, and lifetime compared to this models VBP of $539 K, $1.6 m and $3.6 m for treatment durations of 5 years, 10 years and lifetime. A Budget Impact Model for the US by DeMartino et al. (2021)^49^ concluded that gene therapy for severe SCD is likely to produce a considerable budget impact for Medicaid plans (projected a 1-year budget impact of $29.96 million per state Medicaid program) but may also improve the lives of many and with Wong et al. (2020)^24^ concluding that from a budgetary perspective, universal access to gene therapy should be feasible if taxpayers are willing to pay for it. Lastly, our base case US VBP of $1.6 million is also comparable to the recent ICER analysis’s findings of $1.35–$1.57 million^39^.

Our study has a number of limitations that must be noted. First, we made broad assumptions with respect to short, medium, and long-term metrics of GTx including probabilities of relapse and failure due to a lack of real world evidence. We were also limited to assumptions for the cost of SOC and disability weight, particularly for LMICs; we attempted to plausibly, quantitatively derive these missing estimates indirect means. Similarly, the mortality rate for NBS in this study was based on a single study estimation due to a lack of information in the literature. Given the inherent uncertainties in model parameterization, our results should be viewed as exploratory in nature and, as new information becomes available, they should be reassessed to ensure that appropriate estimates are used.

Second, our model cannot truly represent the clinical outcomes of patients in the remission health state after GTx treatment, who will likely have a predominance of HbS and low-level residual hemolysis and therefore not truly be in “remission”. To address this, future iterations of the model should consider disease weights specific to post-GTx individuals as this information becomes available. Third, we did not evaluate barriers to care for those with SCD, specifically in LMIC, from the perspective of the individual, the provider, or the health care system, where issues of out-of-pocket expenses and lower density of health care centers may limit the roll-out of a potential GTx. Relatedly, we did not evaluate lost productivity and out-of-pocket costs from the perspective of the caregiver. Finally, we did not evaluate costs of development and distribution of GTx, an important component from the perspective of a manufacturer. This paper acknowledges that the VBP for each country is strongly influenced by the country’s incremental cost-effectiveness ratio threshold. Due to the lack of available data for LMIC’s, incremental cost-effectiveness ratio thresholds were calculated based on the Ochelek et al. framework used to reflect the rate at which the healthcare system in each country is able to produce health. Hence, variability in the LMIC thresholds could have some effect on the reported results. Less variability is expected for HIC due to their thresholds being more standardized.

## Conclusion

Our results offer an insight into the VBP for GTx for SCD in a wide range of settings and indicate that a wide range of prices will be required to best maximize access of these therapies to all in need. We hope that this study provides useful input to the development of ‘target product profiles’ of GTx for SCD and to the way in which companies respond to the heterogeneous landscape of burden and affordability. Furthermore, the model can be utilized for supporting and estimating differential pricing across countries in a wide range of potential scenarios for GTx alongside the country specific disease parameters for SCD.

### Supplementary Information

Supplementary Information.

## Data Availability

The datasets generated during and/or analysed during the current study are available from the corresponding author on reasonable request.

## Abbreviations

DALY: Disability-adjusted life-year
GTx: Gene therapy
HIC: High income country
ICER: Incremental cost-effectiveness ratio
LMIC: Low-to-middle-income country
SCD: Sickle cell disease
SOC: Standard of care
VBP: Value-based price
VOC: Vaso-occlusive crisis
