# Supplementary material for: The value-based price of transformative gene therapy for sickle cell disease: a modeling analysis
Source: Sci Rep. 2024 Feb 1;14:2739. doi: 10.1038/s41598-024-53121-0 (PMC10834512; doi:10.1038/s41598-024-53121-0)
Supplement: Supplementary file 1 — Supplementary Information. [file 41598_2024_53121_MOESM1_ESM.docx]

**Supplementary Appendix:**

**Title:** The Value-Based Price of Transformative Gene Therapy for SCD: A Modeling Analysis

Morgan G^1^, Back E^1^, Besser M^2^, Hallett TB^2^, Guzauskas GF^1,4^

1. HCD Economics, The Innovation Centre, Keckwick Lane, Daresbury, WA4 4FS. UK.
2. MRC Centre for Global Infectious Disease Analysis, Imperial College London, London, UK
3. Departments of Haematology, Addenbrooke’s Hospital, Cambridge, UK
4. The Comparative Health Outcomes, Policy, and Economics Institute, University of Washington, Seattle, WA, USA

Contents:

Table A1. Costing Inputs

Table A2. Health State Cost Calculation

Table A3. Country Specific Cost Calculation

Table A4. Congo Cost Comparison

Tabe A5. Model Parameters

Figure A1. Transition probabilities to Mild by Age and Standard of Care Multiplier (0, 5%, 10%)

Figure A2. Transition probabilities to Moderate by Age and Standard of Care Multiplier (0, 5%, 10%)

Figure A3. Transition probabilities to Severe by Age and Standard of Care Multiplier (0, 5%, 10%)

Figure A4. Markov Model Cohort Trace Gene Therapy Arm

Figure A5. Markov Model Cohort Trace Standard of Care Arm

Figure A6 Tornado diagram ranking the parameters that estimate uncertainty in the VBP outcome for Ghana

Figure A7 Tornado diagram ranking the parameters that estimate uncertainty in the VBP outcome for India

Figure A8 Tornado diagram ranking the parameters that estimate uncertainty in the VBP outcome for Kenya

Figure A9. Tornado diagram ranking the parameters that estimate uncertainty in the VBP outcome for Nigeria

Figure A10. Tornado diagram ranking the parameters that estimate uncertainty in the VBP outcome for South Africa

Figure A11. Tornado diagram ranking the parameters that estimate uncertainty in the VBP outcome for Uganda

Figure A12. Tornado diagram ranking the parameters that estimate uncertainty in the VBP outcome for Zambia

Figure A13. Tornado diagram ranking the parameters that estimate uncertainty in the VBP outcome for France

Figure A14. Tornado diagram ranking the parameters that estimate uncertainty in the VBP outcome for Germany

Figure A15. Tornado diagram ranking the parameters that estimate uncertainty in the VBP outcome for Italy

Figure A16. Tornado Tornado diagram ranking the parameters that estimate uncertainty in the VBP outcome for Spain

Figure A17. Tornado diagram ranking the parameters that estimate uncertainty in the VBP outcome for UK

Table A1. Costing Inputs

| Parameter | Default | Source |
| --- | --- | --- |
| Mean total healthcare costs over a 5-Year follow-up ($ USD) | | |
|  |  |  |
| Outpatient Pharmacy | $26,854 | Gallagher et al (2022)^1^ |
| Other Outpatient Services | $80,884 | Gallagher et al (2022)^1^ |
| Outpatient Visits | $6,657 | Gallagher et al (2022)^1^ |
| Emergency Room | $13,873 | Gallagher et al (2022)^1^ |
| Inpatient | $146,874 | Gallagher et al (2022)^1^ |

Table A2. Health State Cost Calculation

| SOC costs | 5-year Average | 1-year Average |
| --- | --- | --- |
| Mild |  |  |
| Outpatient pharmacy | $26,854 |  |
| Other outpatient services | $80,884 |  |
| Outpatient visits | $6,657 |  |
| Total | **$114,395** | **$22,879** |
| Moderate |  |  |
| Outpatient pharmacy | $26,854 |  |
| Other outpatient services | $80,884 |  |
| Outpatient visits | $6,657 |  |
| +16.5% Emergency Room and Inpatient | $26,523 |  |
| Total | **$140,918** | **$28,184** |
| Severe |  |  |
| Outpatient pharmacy | $26,854 |  |
| Other outpatient services | $80,884 |  |
| Outpatient visits | $6,657 |  |
| Emergency Room | $13,873 |  |
| Inpatient | $146,874 |  |
| Total | **$275,142** | **$55,028** |

** SOC: standard of care*

Table A3. Country Specific Cost Calculations

|  | Mild Cost | Moderate Cost | Severe Cost |
| --- | --- | --- | --- |
| Health State Costs | $22,879 | $30,916 | $55,028 |
| Country specific costs | $\frac{Country HE}{US HE}\times22,879$ | $\frac{Country HE}{US HE}\times30,916$ | $\frac{Country HE}{US HE}\times55,028$ |

**HE- health expenditure*

Table A4. Congo^2^ Cost Comparison

| Severity | Congo Costs* Ngolet et al. (2016)** (USD) | Ghana- Model Costs (USD) | India - Model Costs (USD) | Kenya - Model Costs (USD) | Nigeria - Model Costs (USD) | Uganda- Model Costs (USD) | Zambia - Model Costs (USD) |
| --- | --- | --- | --- | --- | --- | --- | --- |
| Mild | $111.67 | $157.12 | $134.08 | $173.88 | $148.74 | $67.04 | $144.55 |
| Moderate | $240.355 | $193.55 | $165.17 | $214.20 | $183.23 | $82.58 | $178.07 |
| Severe | $369.04 | $377.90 | $322.48 | $418.21 | $357.75 | $161.24 | $347.67 |

**Mild State (based on hospitalisation, diagnostic tests and medicine costs), Moderate State (Mild state plus adding 1.5 of a VOC), Severe State (Mild state plus adding 3 of a VOC)*

***Ngolet, L. O. et al. Sickle-Cell Disease Healthcare Cost in Africa: Experience of the Congo. Anemia 2016, (2016).*

Table A5. Model Parameters

| **Parameter** | **Default** | **< Sensitivity Range >** | | **Source** |
| --- | --- | --- | --- | --- |
| **Gene Therapy Parameters** | | | | |
| One-Time Gene Therapy Cost (placeholder) | $100,000 |  |  | Assumption |
| Complete Remission Probability | 65% | 35% | 95% | Assumption |
| Relapse Possibility | TRUE | TRUE | FALSE | Assumption |
| └ Remission Durability (years) | 10 | 5 | Lifetime | Assumption |
| Gene Therapy Minimum Age (years) | 12 | 18 | 0 | Assumption |
| Gene Therapy Maximum Age (years) | 50 | 50 | 50 | Assumption |
| Maximum Stage Eligibility for Gene Therapy | Severe | Mild | Severe | Assumption |
| Repeat Gene Therapy After Initial relapse? | FALSE | FALSE | TRUE | Assumption |
| Repeat Gene Therapy After Initial Failure? | FALSE | FALSE | TRUE | Assumption |
| Maximum Annual Gene Therapy Uptake | 50% | 25% | 75% | Assumption |
| └ Ramp Up Time to Maximum Uptake (years) | 10 | 15 | 5 | Assumption |
|  |  |  |  |  |
| **Health Expenditure per capita** | | | | |
| Ghana | $75 | $60 | $90 | Worldbank, 2019^3^ |
| India | $64 | $51 | $77 |  |
| Kenya | $83 | $66 | $100 |  |
| Nigeria | $71 | $57 | $85 |  |
| South Africa | $547 | $438 | $656 |  |
| Uganda | $32 | $26 | $38 |  |
| Zambia | $69 | $55 | $83 |  |
| France | $4,492 | $3,594 | $5,390 |  |
| Germany | $5,440 | $4,352 | $6,528 |  |
| Italy | $2,906 | $2,325 | $3,487 |  |
| Spain | $2,711 | $2,169 | $3,253 |  |
| UK | $4,313 | $3,450 | $5,176 |  |
| USA | $10,921 | $8,737 | $13,105 |  |
|  |  |  |  |  |
| **Health Expenditure per capita as a proportion (US Base)** | | | | |
| Ghana | 1.00% | 0.80% | 1.20% | Calculated. Calculation based on country-specific health Expenditure (US$) per capita related to USA health expenditure per capita  Calculated. |
| India | 1.00% | 0.80% | 1.20% |  |
| Kenya | 1.00% | 0.80% | 1.20% |  |
| Nigeria | 1.00% | 0.80% | 1.20% |  |
| South Africa | 5.00% | 4.00% | 6.00% |  |
| Uganda | 0.00% | 0.00% | 0.00% |  |
| Zambia | 1.00% | 0.80% | 1.20% |  |
| France | 41.00% | 32.80% | 49.20% |  |
| Germany | 50.00% | 40.00% | 60.00% |  |
| Italy | 27.00% | 21.60% | 32.40% |  |
| Spain | 25.00% | 20.00% | 30.00% |  |
| UK | 39.00% | 31.20% | 46.80% |  |
| USA | 100.00% | 80.00% | 120.00% |  |
|  |  |  |  |  |
| **SCD Costs** | | | | |
| **Annual Mild cost** |  |  |  |  |
| Ghana | $157 | $126 | $189 | Calculated (Country Health Expenditure per capita / US Health Expenditure per capita) multiplied by cost of a mild VOC. Calculation based on "Proportion (US base) from Ghana" and cost of a mild VOC. |
| India | $134 | $107 | $161 |  |
| Kenya | $174 | $139 | $209 |  |
| Nigeria | $149 | $119 | $178 |  |
| South Africa | $1,146 | $917 | $1,375 |  |
| Uganda | $67 | $54 | $80 |  |
| Zambia | $145 | $116 | $173 |  |
| France | $9,411 | $7,528 | $11,293 |  |
| Germany | $11,397 | $9,117 | $13,676 |  |
| Italy | $6,088 | $4,870 | $7,306 |  |
| Spain | $5,679 | $10,928 | $16,392 |  |
| UK | $9,036 | $7,228 | $10,843 |  |
| USA | $22,879 | $18,303 | $27,455 |  |
|  |  |  |  |  |
| **Annual Moderate Cost** |  |  |  |  |
| Ghana | $194 | $155 | $232 | Calculated (Country Health Expenditure per capita / US Health Expenditure per capita) multiplied by cost of a moderate VOC. |
| India | $165 | $132 | $198 |  |
| Kenya | $214 | $171 | $257 |  |
| Nigeria | $183 | $147 | $220 |  |
| South Africa | $1,412 | $1,129 | $1,694 |  |
| Uganda | $83 | $66 | $99 |  |
| Zambia | $178 | $142 | $214 |  |
| France | $11,593 | $9,274 | $13,911 |  |
| Germany | $14,039 | $11,231 | $16,847 |  |
| Italy | $7,500 | $6,000 | $8,999 |  |
| Spain | $6,996 | $5,597 | $8,396 |  |
| UK | $11,131 | $8,905 | $13,357 |  |
| USA | $28,184 | $22,547 | $33,821 |  |
|  |  |  |  |  |
| **Annual Severe Cost** |  |  |  |  |
| Ghana | $378 | $302 | $453 | Calculated (Country Health Expenditure per capita / US Health Expenditure per capita) multiplied by cost of a severe VOC. |
| India | $322 | $258 | $387 |  |
| Kenya | $418 | $335 | $502 |  |
| Nigeria | $358 | $286 | $429 |  |
| South Africa | $2,756 | $2,205 | $3,307 |  |
| Uganda | $161 | $129 | $193 |  |
| Zambia | $348 | $278 | $417 |  |
| France | $22,634 | $18,107 | $27,161 |  |
| Germany | $27,411 | $21,929 | $32,893 |  |
| Italy | $14,643 | $11,714 | $17,571 |  |
| Spain | $13,660 | $10,928 | $16,392 |  |
| UK | $21,732 | $17,386 | $26,078 |  |
| USA | $55,028 | $44,022 | $66,034 |  |
|  |  |  |  |  |
| **Annual Societal Cost Mild** |  |  |  |  |
| Ghana | $39 | $31 | $47 | Calculated (Country Health Expenditure per capita multiplied by 0.517 (0.517 comes from the Rizio et al, 2020 paper. It is the percentage productivity loss by VOC severity (51.7%) for those who are classed as less severe, in our case Mild or Moderate)^4^ |
| India | $33 | $26 | $40 |  |
| Kenya | $43 | $34 | $51 |  |
| Nigeria | $37 | $29 | $44 |  |
| South Africa | $283 | $226 | $339 |  |
| Uganda | $17 | $13 | $20 |  |
| Zambia | $36 | $29 | $43 |  |
| France | $2,322 | $1,858 | $2,787 |  |
| Germany | $2,812 | $2,250 | $3,375 |  |
| Italy | $1,502 | $1,202 | $1,803 |  |
| Spain | $1,402 | $1,121 | $1,682 |  |
| UK | $2,230 | $1,784 | $2,676 |  |
| USA | $5,646 | $4,517 | $6,775 |  |
|  |  |  |  |  |
| **Societal Cost Moderate** |  |  |  |  |
| Ghana | $39 | $31 | $47 | Calculated (Country Health Expenditure per capita multiplied by 0.517 (0.517 comes from the Rizio et al, 2020 paper. It is the percentage productivity loss by VOC severity (51.7%) for those who are classed as less severe, in our case Mild or Moderate)^4^ |
| India | $33 | $26 | $40 |  |
| Kenya | $43 | $34 | $51 |  |
| Nigeria | $37 | $29 | $44 |  |
| South Africa | $283 | $226 | $339 |  |
| Uganda | $17 | $13 | $20 |  |
| Zambia | $36 | $29 | $43 |  |
| France | $2,322 | $1,858 | $2,787 |  |
| Germany | $2,812 | $2,250 | $3,375 |  |
| Italy | $1,502 | $1,202 | $1,803 |  |
| Spain | $1,402 | $1,121 | $1,682 |  |
| UK | $2,230 | $1,784 | $2,676 |  |
| USA | $5,646 | $4,517 | $6,775 |  |
|  |  |  |  |  |
| **Societal Cost Severe** |  |  |  |  |
| Ghana | $47 | $37 | $56 | Calculated (Country Health Expenditure per capita multiplied by 0.621. 0.621 comes from the Rizio et al, 2020 paper. It is the percentage productivity loss by VOC severity (62.1%) for those who are classed as Severe.^4^ |
| India | $40 | $32 | $48 |  |
| Kenya | $52 | $41 | $62 |  |
| Nigeria | $44 | $35 | $53 |  |
| South Africa | $340 | $272 | $408 |  |
| Uganda | $20 | $16 | $24 |  |
| Zambia | $43 | $34 | $51 |  |
| France | $2,790 | $2,232 | $3,347 |  |
| Germany | $3,378 | $2,703 | $4,054 |  |
| Italy | $1,805 | $1,444 | $2,166 |  |
| Spain | $1,684 | $1,347 | $2,020 |  |
| UK | $2,678 | $2,143 | $3,214 |  |
| USA | $6,782 | $5,426 | $8,138 |  |
|  |  |  |  |  |
| **Proportion distribution at cycle 0** | | | | |
| Proportion female | 51% | 41% | 61% | Salcedo et al., 2021^5^ |
| Proportion Newborn Screened LMIC | 5% | 4% | 6% | Oron et al, 2020^6^ |
| Proportion Newborn Screened HIC | 97% | 78% | 100% | Streetly et al, 2017^7^ |
|  |  |  |  |  |
| **SCD Severity** | | | | |
| Mild SCD (calculated) | 50% | 40% | 60% | Calculated (AVG male with mild SCD + AVG female with mild SCD) ÷ 2 = (48.3% + 50.8%) ÷ 2 = 49.55% **(50%)**. Calculation based on Salcedo et al., 2021.^5^ |
| Moderate SCD | 23% | 18% | 27% | Calculated (AVG male with moderate SCD + AVG female with moderate SCD) ÷ 2 = (22.1% + 23.1%) ÷ 2 = 22.6% **(23%).** Calculation based on Salcedo et al., 2021.^5^ |
| Severe SCD | 28% | 22% | 33% | Calculated (AVG male with severe SCD + AVG female with severe SCD) ÷ 2 = (27.1% + 28.7%) ÷ 2 = 27.9% **(28%)**. Calculation based on Salcedo et al., 2021.^5^ |
|  |  |  |  |  |
| **SCD Mortality** | | | | |
| **Relative Risk of Death With Newborn Screening (ORs converted to RRs)** |  |  |  |  |
| Age 0 | 0.010 | 0.030 | 0.300 | Inputs were converted from Odds Ratio to Risk Ratio. Calculation: (1-EXP(-X)) based on mortality rate of the treatment group. Calculation based on Runkel et al, 2020^8^ |
| Age 1 | 0.010 | 0.020 | 0.200 |  |
| Age 2-3 | 0.010 | 0.020 | 0.150 |  |
| Age 4-8 | 0.020 | 0.040 | 0.220 |  |
| Age 9+ | 0.086 | 0.070 | 1.640 |  |
|  |  |  |  |  |
| **Annual Probability of Death without Newborn Screening** |  |  |  |  |
| Age 0 | 0.095 | 0.039 | 0.139 | Calculated. Calculation (1-EXP(-X)) based on mortality rate of the control group. Calculation based on Runkel et al, 2020^8^ |
| Age 1 | 0.131 | 0.068 | 0.181 |  |
| Age 2-3 | 0.156 | 0.095 | 0.221 |  |
| Age 4-8 | 0.173 | 0.113 | 0.237 |  |
| Age 9+ | 0.205 | 0.139 | 0.274 |  |
|  |  |  |  |  |
| **Disability Weights** | | | | |
| Unaffected / Remission via Gene Therapy | 0.000 | 0.000 | 0.000 |  |
| Mild SCD | 0.004 | 0.001 | 0.008 | Salomon et al, 2015^9^ |
| Moderate SCD | 0.052 | 0.034 | 0.076 |  |
| Severe SCD | 0.149 | 0.101 | 0.209 |  |
|  |  |  |  |  |
| **Life Expectancy (years)** | | | | |
| Ghana | 69.37 | 55 | 83 | [CIA:](https://www.cia.gov/the-world-factbook/countries/ghana/) The World Factbook^10^ |
| India | 67.22 | 54 | 81 |  |
| Kenya | 69.69 | 56 | 84 |  |
| Nigeria | 61.33 | 49 | 74 |  |
| South Africa | 69.32 | 55 | 83 |  |
| Uganda | 68.96 | 55 | 83 |  |
| Zambia | 66.26 | 53 | 80 |  |
| France | 82.59 | 66 | 99 |  |
| Germany | 81.51 | 65 | 98 |  |
| Italy | 82.59 | 66 | 99 |  |
| Spain | 82.55 | 66 | 99 |  |
| UK | 81.94 | 66 | 98 |  |
| USA | 80.59 | 64 | 97 |  |

Figure A1. Transition probabilities to Mild by Age and Standard of Care Multiplier (0, 5%, 10%)


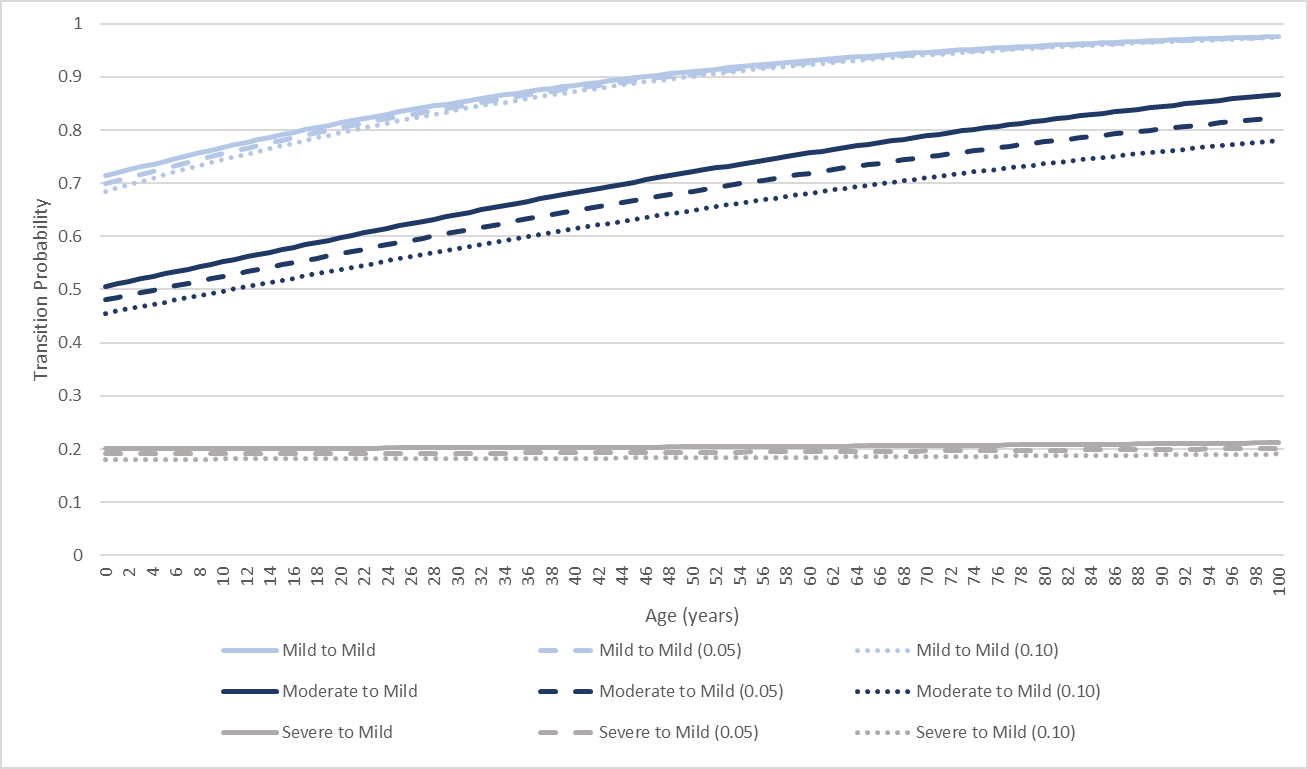


Figure A2 Transition probabilities to Moderate by Age and Standard of Care Multiplier (0, 5%, 10%)


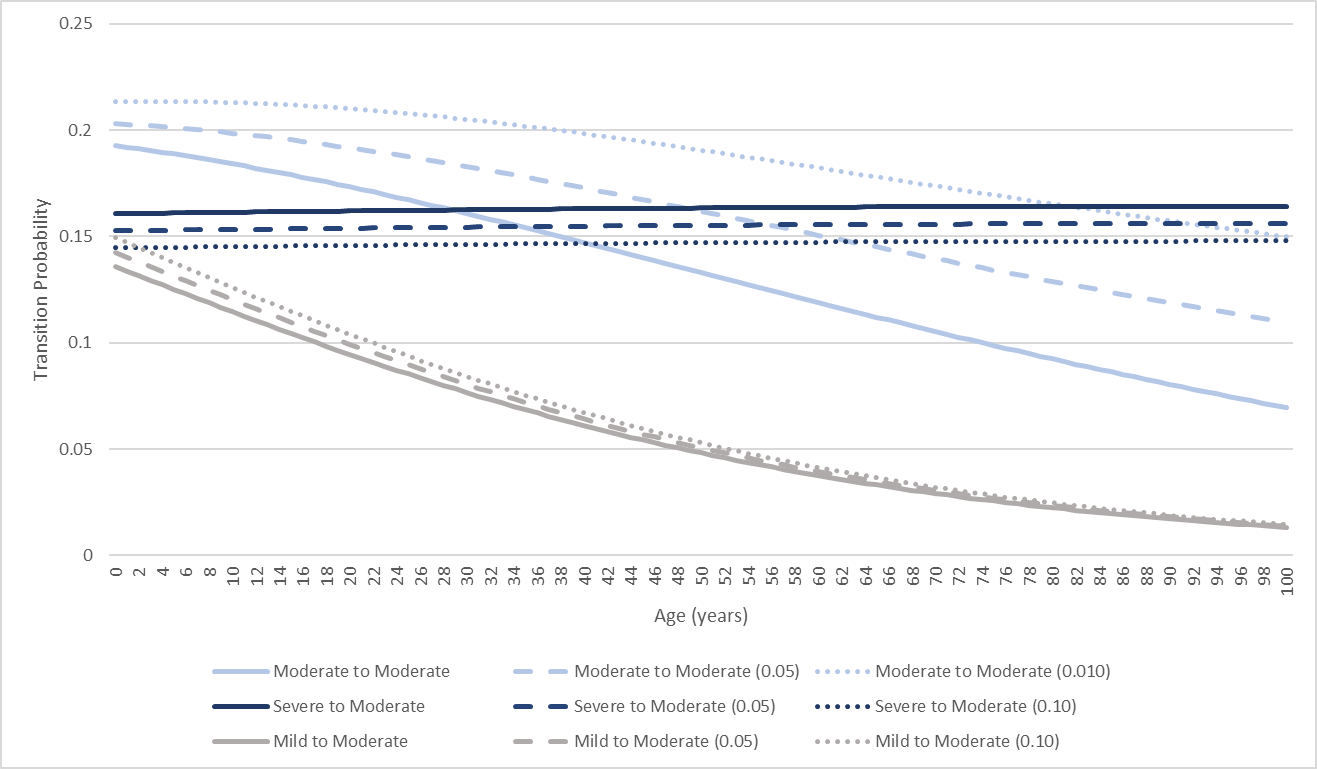


Figure A3 Transition probabilities to Severe by Age and Standard of Care Multiplier (0, 5%, 10%)


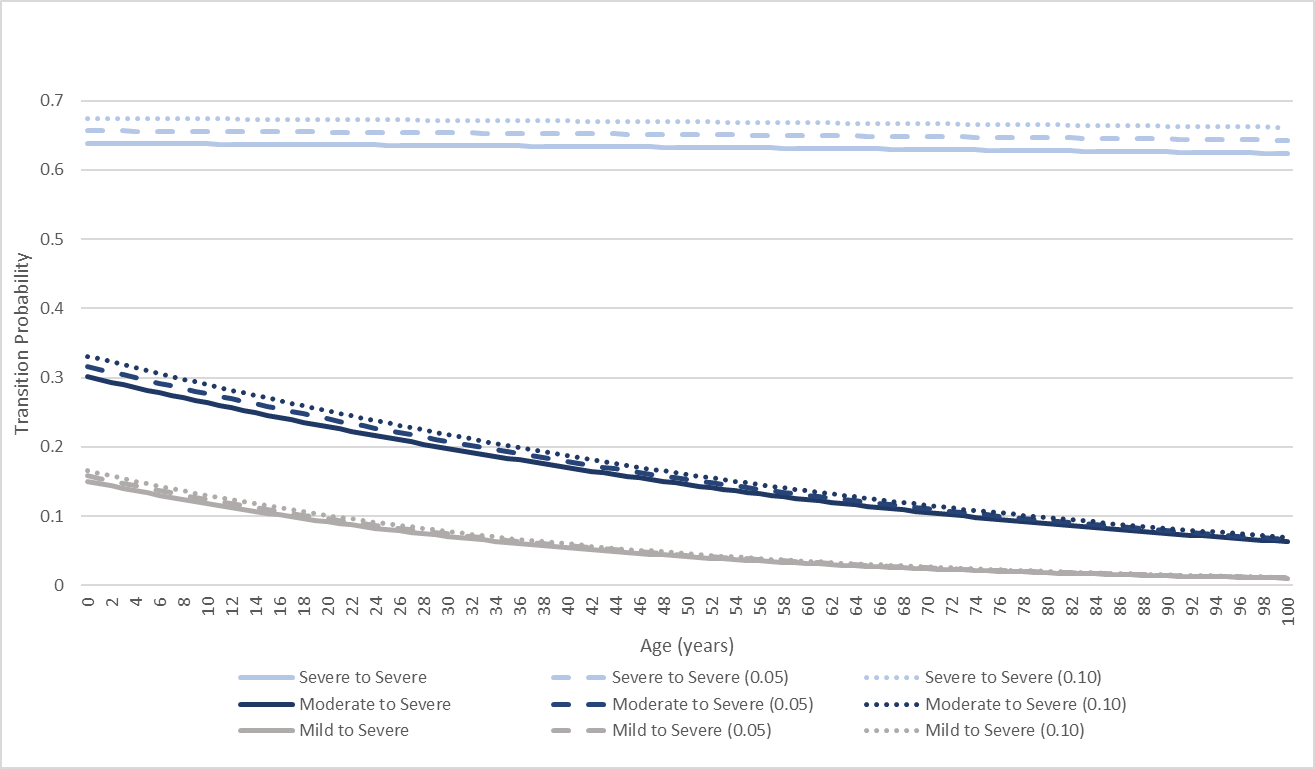


Figure A4. Markov Model Cohort Trace Gene Therapy Arm


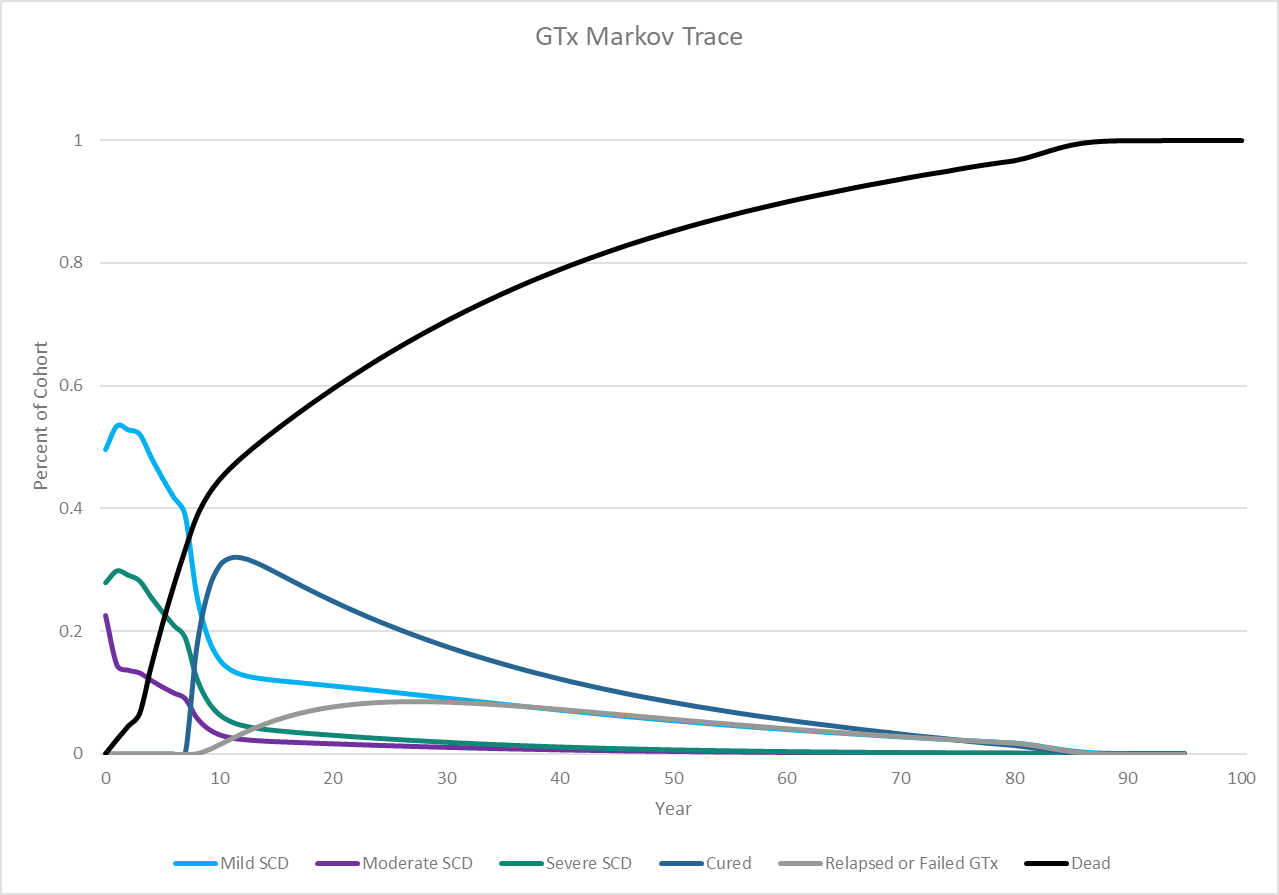


Figure A5. Markov Model Cohort Trace Standard of Care Arm


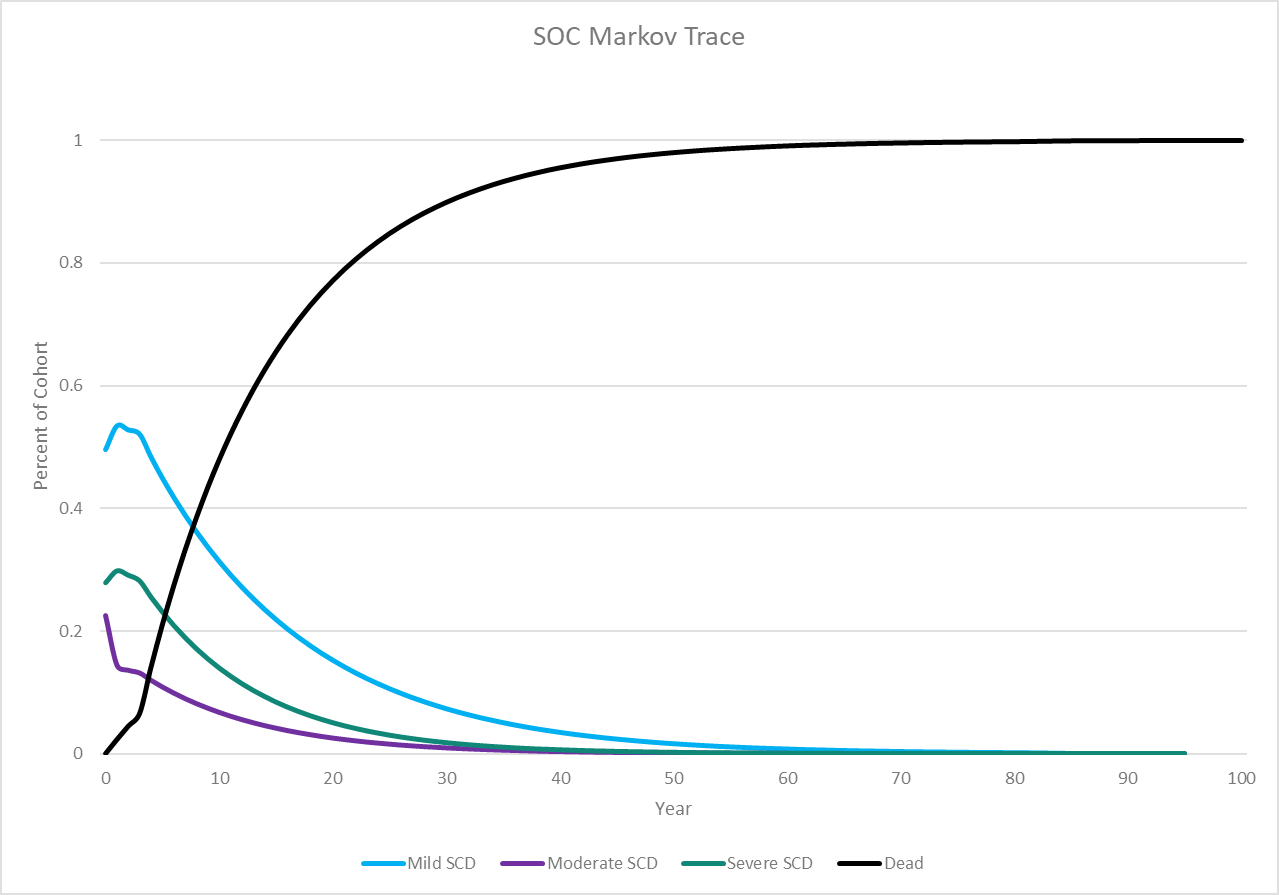


Figure A6 Tornado diagram ranking the parameters that estimate uncertainty in the VBP outcome for Ghana


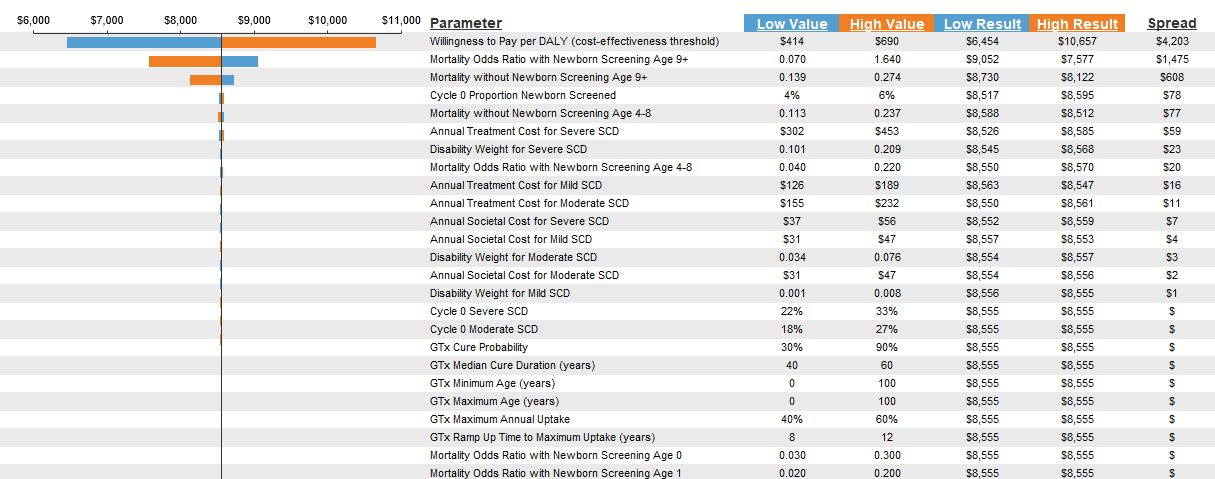


Figure A7 Tornado diagram ranking the parameters that estimate uncertainty in the VBP outcome for India


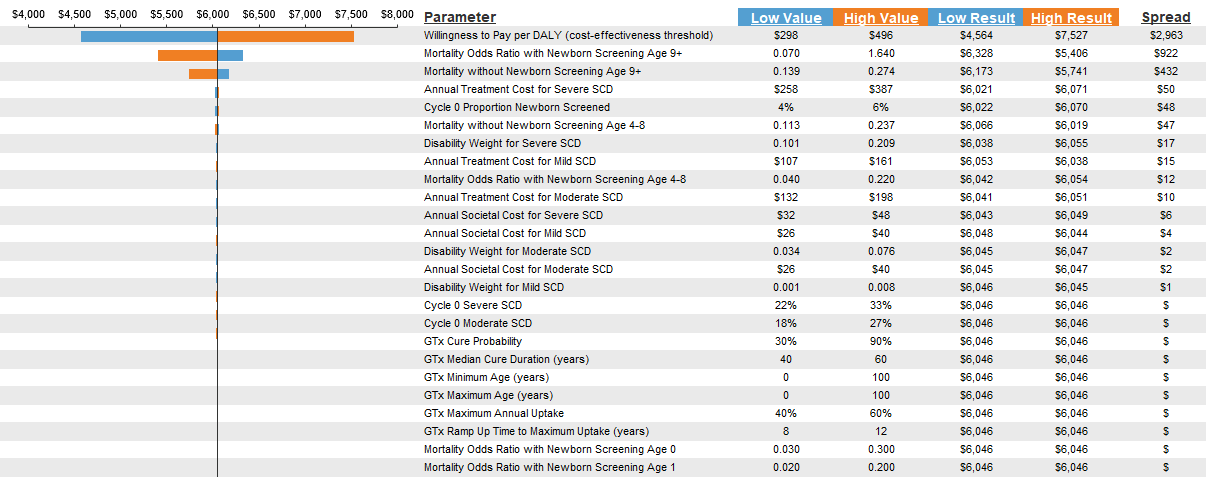


Figure A8 Tornado diagram ranking the parameters that estimate uncertainty in the VBP outcome for Kenya


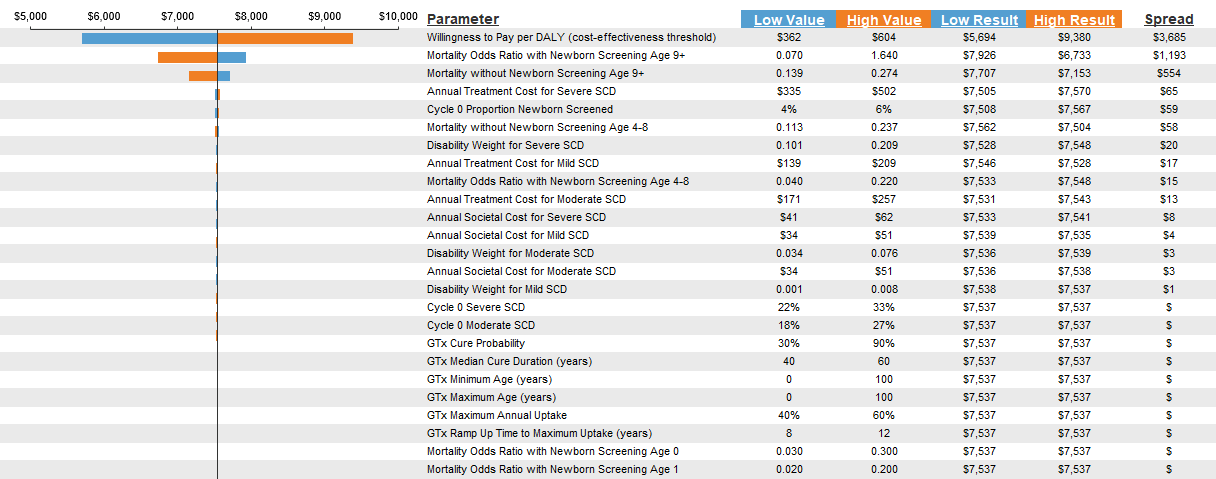


Figure A9 Tornado diagram ranking the parameters that estimate uncertainty in the VBP outcome for Nigeria


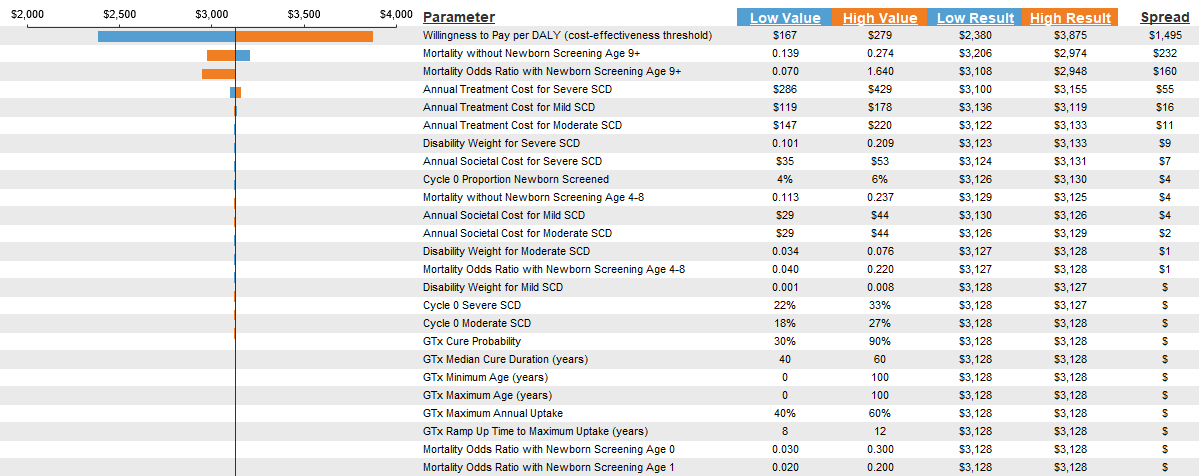


Figure A10 Tornado diagram ranking the parameters that estimate uncertainty in the VBP outcome for South Africa


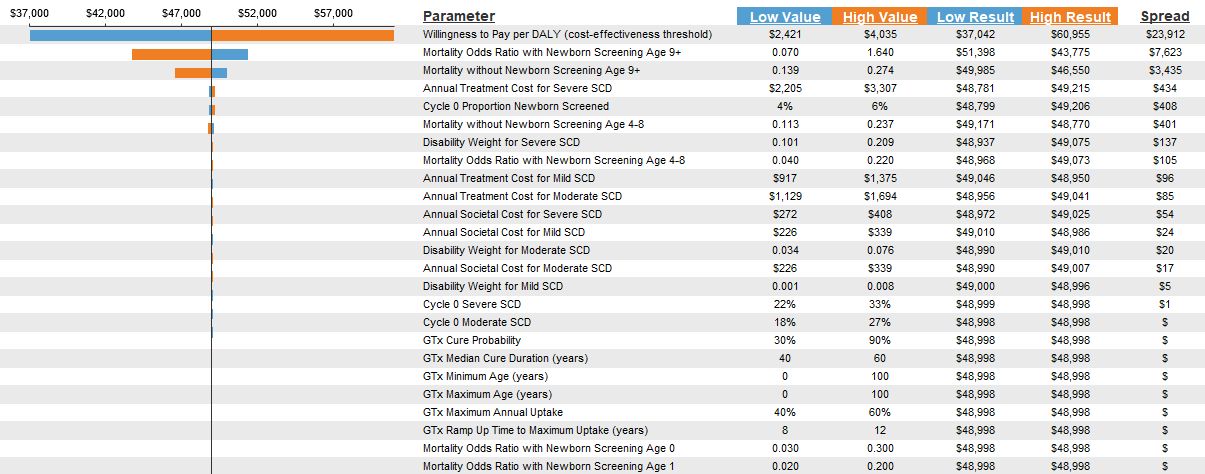


Figure A11 Tornado diagram ranking the parameters that estimate uncertainty in the VBP outcome for Uganda


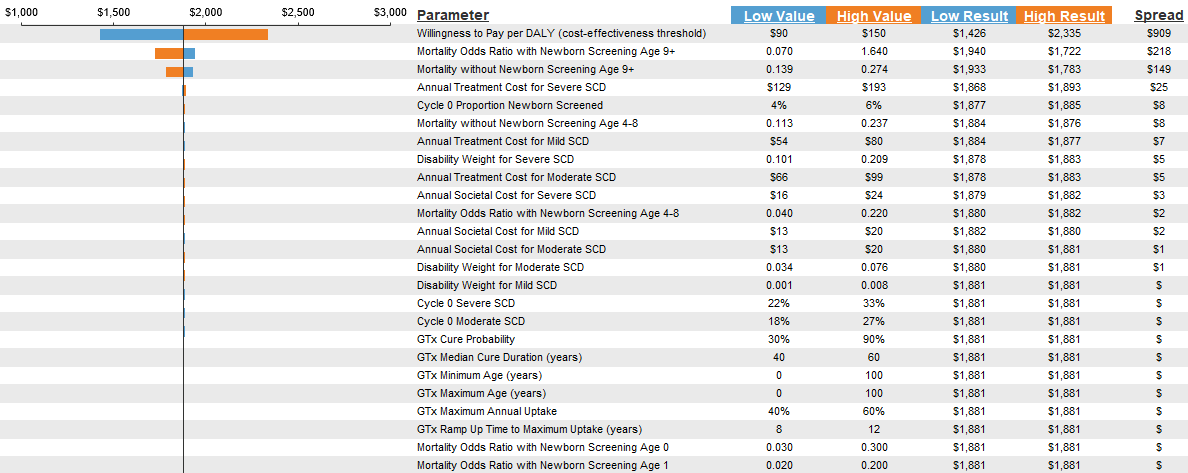


Figure A12 Tornado diagram ranking the parameters that estimate uncertainty in the VBP outcome for Zambia


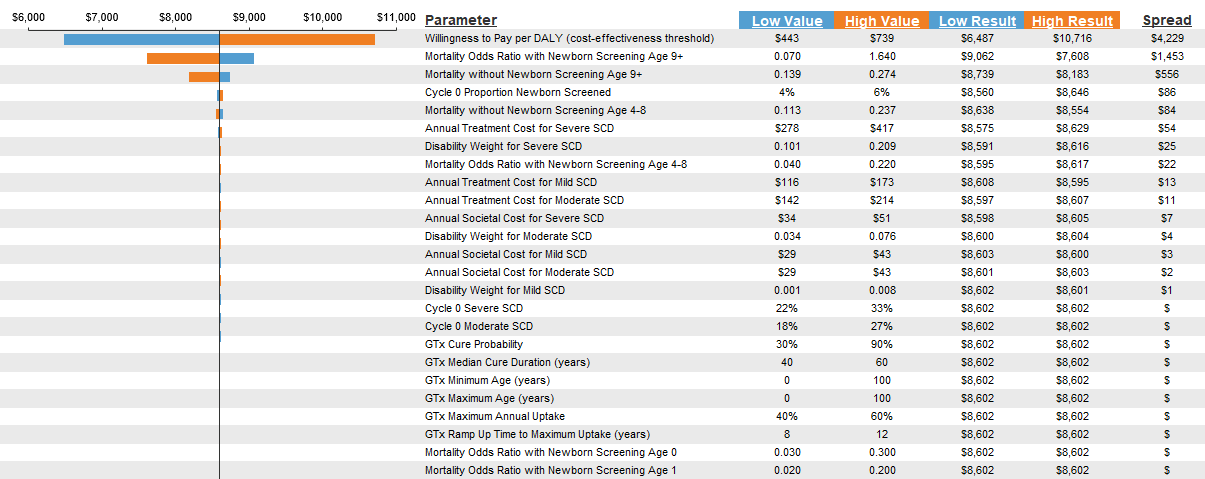


Figure A13 Tornado diagram ranking the parameters that estimate uncertainty in the VBP outcome for France


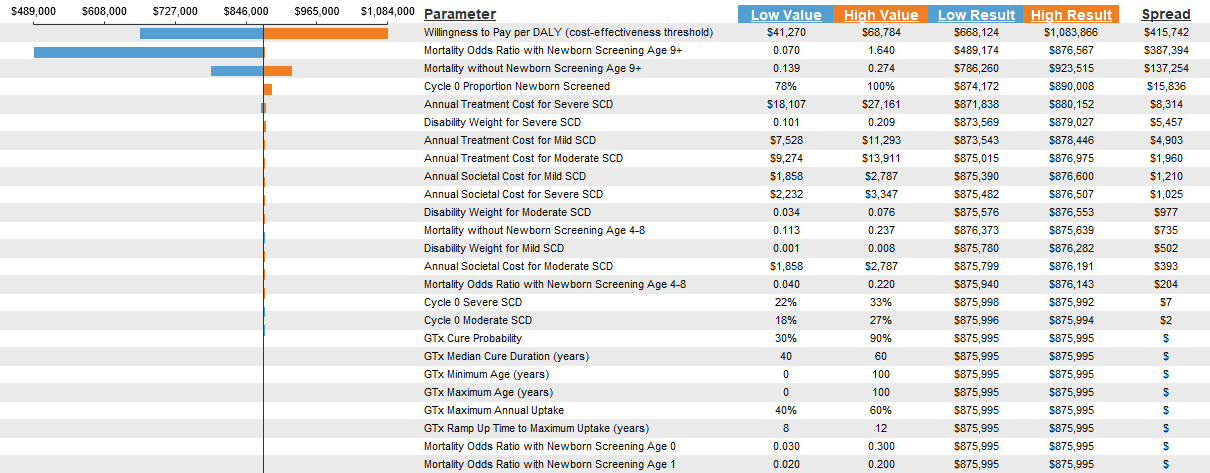


Figure A14 Tornado diagram ranking the parameters that estimate uncertainty in the VBP outcome for Germany


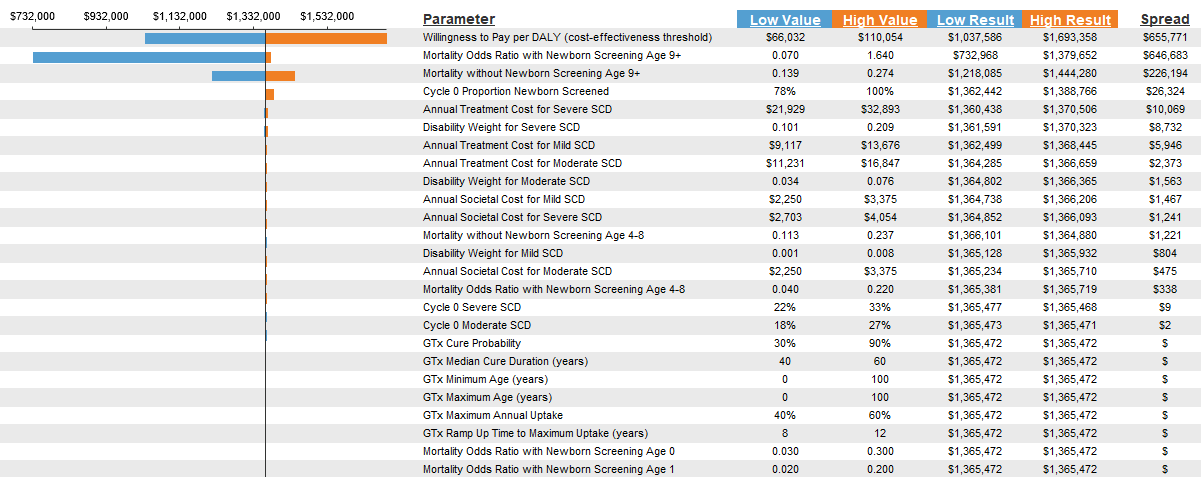


Figure A15 Tornado diagram ranking the parameters that estimate uncertainty in the VBP outcome for Italy


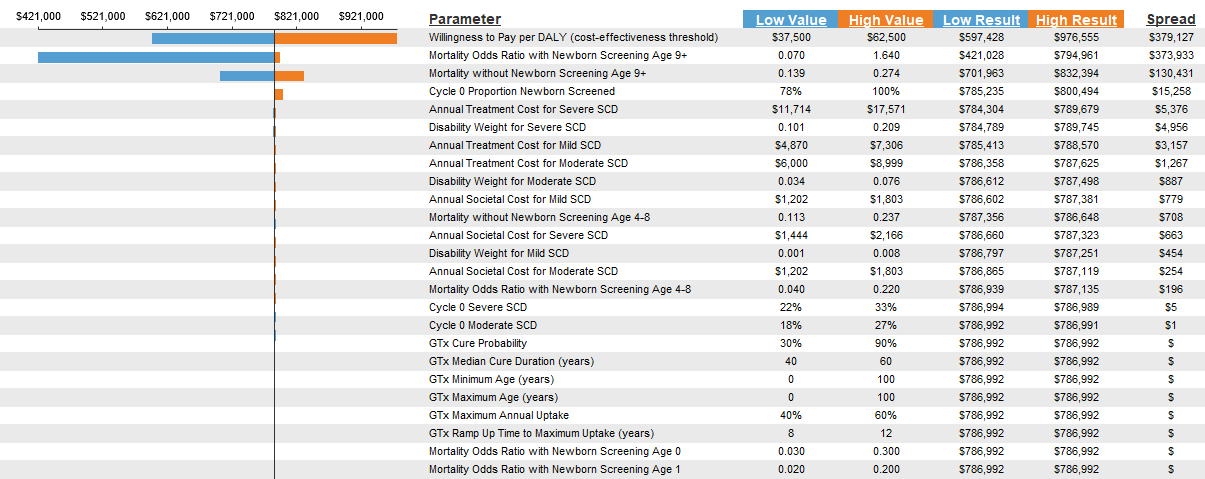


Figure A16 Tornado diagram ranking the parameters that estimate uncertainty in the VBP outcome for Spain


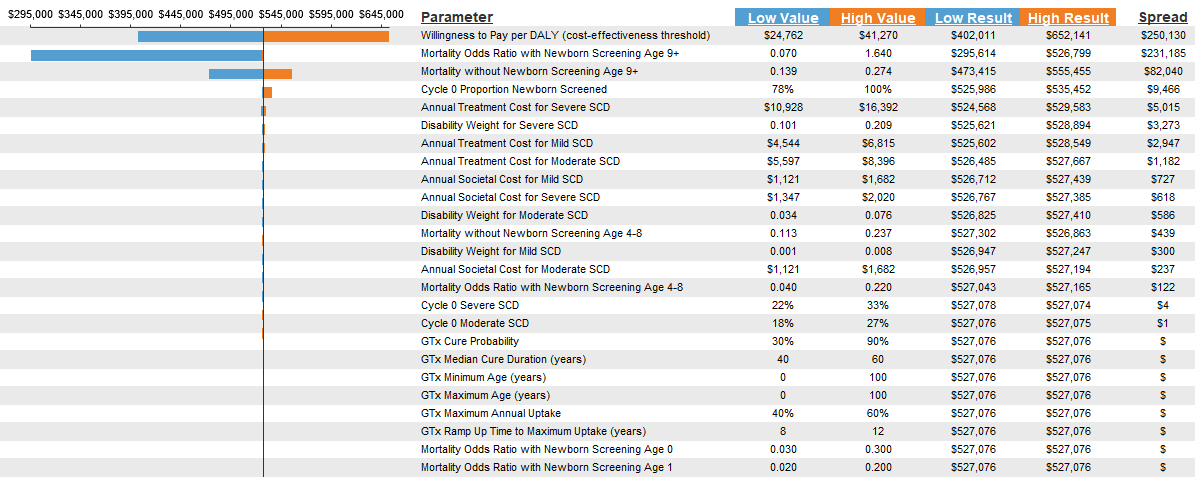


Figure A17 Tornado diagram ranking the parameters that estimate uncertainty in the VBP outcome for UK


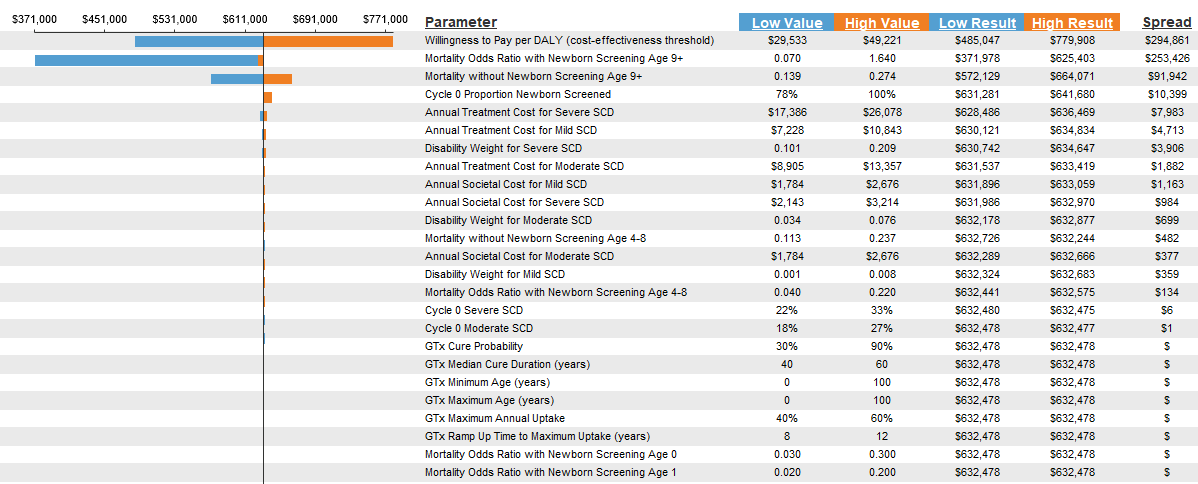


Supplement References

1. Gallagher ME, Chawla A, Brady BL, Badawy SM. Heterogeneity of the long-term economic burden of severe sickle cell disease: a 5-year longitudinal analysis. *J Med Econ*. 2022;25(1):1140-1148. doi:10.1080/13696998.2022.2133824

2. Ngolet LO, Moyen Engoba M, Kocko I, Elira Dokekias A, Mombouli JV, Moyen GM. Sickle-Cell Disease Healthcare Cost in Africa: Experience of the Congo. *Anemia*. 2016;2016. doi:10.1155/2016/2046535

3. World Bank Open Data | Data. Accessed February 1, 2023. https://data.worldbank.org/

4. Rizio AA, Bhor M, Lin X, et al. The relationship between frequency and severity of vaso-occlusive crises and health-related quality of life and work productivity in adults with sickle cell disease. *Qual Life Res*. 2020;29(6):1533-1547. doi:10.1007/S11136-019-02412-5

5. Salcedo J, Bulovic J, Young CM. Cost-effectiveness of a hypothetical cell or gene therapy cure for sickle cell disease. *Scientific Reports 2021 11:1*. 2021;11(1):1-13. doi:10.1038/s41598-021-90405-1

6. Oron AP, Chao DL, Ezeanolue EE, et al. Caring for Africa’s sickle cell children: Will we rise to the challenge? *BMC Med*. 2020;18(1):1-8. doi:10.1186/S12916-020-01557-2/FIGURES/1

7. Streetly A, Sisodia R, Dick M, Latinovic R, Hounsell K, Dormandy E. Evaluation of newborn sickle cell screening programme in England: 2010-2016. *Arch Dis Child*. 2018;103(7):648-653. doi:10.1136/ARCHDISCHILD-2017-313213

8. Runkel B, Klüppelholz B, Rummer A, et al. Screening for sickle cell disease in newborns: a systematic review. *Syst Rev*. 2020;9(1). doi:10.1186/S13643-020-01504-5

9. Salomon JA, Haagsma JA, Davis A, et al. Disability weights for the Global Burden of Disease 2013 study. *Lancet Glob Health*. 2015;3(11):e712-e723. doi:10.1016/S2214-109X(15)00069-8

10. https://www.cia.gov/the-world-factbook/countries/. CIA: The World Factbook.
